# Complete Genome Sequence and Methylome Analysis of Bacillus Strain X1

**DOI:** 10.1128/genomeA.01593-14

**Published:** 2015-02-19

**Authors:** Alexey Fomenkov, Keith D. Lunnen, Zhenyu Zhu, Brian P. Anton, Geoffrey G. Wilson, Tamas Vincze, Richard J. Roberts

**Affiliations:** New England Biolabs, Ipswich, Massachusetts, USA

## Abstract

Bacillus strain X1 is the source strain for the restriction enzyme BstXI. Its complete sequence and full methylome was determined using single-molecule real-time (SMRT) sequencing.

## GENOME ANNOUNCEMENT

Bacillus strain X1 *(*originally called Bacillus stearothermophilus X1) was originally provided by Dr. Neil Welker, while he was at Northwestern University, and is now housed in the New England Biolabs culture collection (NEB278). It is the original source of the type II restriction enzyme (REase) BstXI, first isolated in 1981 and sold commercially in 1983 (1). This enzyme recognizes and cleaves the bipartite DNA sequence CCAN↓NNNNNTGG. The complete genome sequence for Bacillus strain XI was determined using a Pacific Biosciences (PacBio, Menlo Park, CA) RSII instrument (2). Three single-molecule real-time (SMRT) cells were used, generating 1,100 Mb of sequence which was assembled into a closed circular genome of 3,422,674 bp (>300 fold coverage). Using the SMRTPortal 2.2 analysis platform, two symmetrically methylated sequence motifs were detected, CC**^6m^**ANNNNNNTGG, corresponding to the recognition sequence for M.BstXI, and G**^6m^**ATC, which corresponded to the methylation target of the three BstXII restriction-modification system methylases (**^6m^**A = *N*6-methyladenine). The genes responsible for these modifications were identified by amino acid sequence comparisons with previously characterized DNA methyltransferases (MTases) as described previously (3). HW35_9765 and HW35_09760 encode the modification and specificity subunits of a typical type I MTase which, characteristically, modify bipartite DNA sequences. Subcloning into Escherichia coli confirmed that these code for the M.BstXI MTase, and that a nearby gene, HW35_9755, codes for the BstXI REase. The complete system is shown schematically in REBASE (http://rebase.neb.com/cgi-bin/seqget?BstXI) (1). Restriction modification (R-M) systems composed of a type II REase partnered with type I MTase have been found previously (4). M.BstXI is unusual, however, because its specificity subunit contains two recognition domains yet recognizes a sequence that is symmetric overall, implying that both domains recognize the same, 5′-CCA-3′, half-sequence. These domains display close similarity to each other and to the single recognition domain of M.XcmI, which methylates the related sequence, CC**^6m^**ANNNNNNNNNTGG. Also present in the genome is a more complicated R-M system comprising one REase gene (HW35_01470) and three MTase genes (REBASE: http://rebase.neb.com/cgi-bin/seqget?BstXII). All four encode proteins with strong similarity to well-characterized enzymes recognizing GATC (5). Subcloning demonstrated that these genes are active, although only that encoding M2.BstXII resulted in complete modification. One additional gene, HW35_12570, encoded a protein (BstORFCP), with significant similarity to a family of methyl-dependent REases found in Staphylococcus aureus (e.g., SauUSI, which recognizes S^5m^CNGS) (6).

### Nucleotide sequence accession number.

This complete genome sequence has been deposited in DDBJ/ENA/GenBank under the accession no. CP008855 (BioProject PRJNA253895).
